# Supplementary material for: Impact of natural disasters on HIV risk behaviors, seroprevalence, and virological supression in a hyperendemic fishing village in Uganda
Source: PLoS One. 2024 Oct 11;19(10):e0293711. doi: 10.1371/journal.pone.0293711 (PMC11469503; doi:10.1371/journal.pone.0293711)
Supplement: S1 Table — (DOCX) [file pone.0293711.s001.docx]

**S1 Table.** **Summary of participation and non-participation in the pre- and post-COVID periods.**

| **Survey period** | **Participated** | **Away for work or school** | **Refused** |
| --- | --- | --- | --- |
| **Overall** |  |  |  |
| Pre-COVID | 2209 (50.6) | 2154 (49.3) | 3 (0.1) |
| Post-COVID | 2756 (60.9) | 1682 (37.1) | 91 (2) |
| **Male** |  |  |  |
| Pre-COVID | 1181 (46.6) | 1348 (53.2) | 3 (0.1) |
| Post-COVID | 1523 (57.9) | 1058 (40.2) | 50 (1.9) |
| **Female** |  |  |  |
| Pre-COVID | 1028 (56.1) | 806 (43.9) | 0 (0) |
| Post-COVID | 1233 (65) | 624 (32.9) | 41 (2.2) |
