# Supplementary material for: Impact of natural disasters on HIV risk behaviors, seroprevalence, and virological supression in a hyperendemic fishing village in Uganda
Source: PLoS One. 2024 Oct 11;19(10):e0293711. doi: 10.1371/journal.pone.0293711 (PMC11469503; doi:10.1371/journal.pone.0293711)
Supplement: S2 Table — (DOCX) [file pone.0293711.s002.docx]

**S2 Table. Reasons for lost to follow-up in the pre- and post-COVID periods.**

| **Survey period** | **Away for work or school** | **Refused** | **Other** | **Already seen** | **Out- migrated** | **Dead** | **Ineligible** |
| --- | --- | --- | --- | --- | --- | --- | --- |
| **Overall** |  |  |  |  |  |  |  |
| Pre-COVID | 2154 (62.6) | 3 (0.1) | 10 (0.3) | 13 (0.4) | 1201(34.9) | 36 (1) | 25 (0.7) |
| Post-COVID | 1682 (46.9) | 91 (2.5) | 19 (0.5) | 21(0.6) | 1654 (46.1) | 84 (2.3) | 34 (0.9) |
| **Male** |  |  |  |  |  |  |  |
| Pre-COVID | 1348 (67.9) | 3 (0.2) | 6 (0.3) | 13 (0.7) | 571 (28.8) | 24 (1.2) | 19 (1) |
| Post-COVID | 1058 (52.7) | 50 (2.5) | 11 (0.5) | 16 (0.8) | 797 (39.7) | 51 (2.5) | 23 (1.1) |
| **Female** |  |  |  |  |  |  |  |
| Pre-COVID | 806 (55.3) | 0 (0) | 4 (0.3) | 0 (0) | 630 (43.2) | 12 (0.8) | 6 (0.4) |
| Post-COVID | 624 (39.5) | 41 (2.6) | 8 (0.5) | 5(0.3) | 857 (54.3) | 33 (2.1) | 11 (0.7) |
