# Supplementary material for: Impact of natural disasters on HIV risk behaviors, seroprevalence, and virological supression in a hyperendemic fishing village in Uganda
Source: PLoS One. 2024 Oct 11;19(10):e0293711. doi: 10.1371/journal.pone.0293711 (PMC11469503; doi:10.1371/journal.pone.0293711)
Supplement: S3 Table — (DOCX) [file pone.0293711.s003.docx]

**S3 Table. Comparison of baseline characteristics of respondents who were and were not lost to follow-up between the two RCCS surveys.**

|  | **Participated in post-COVID-19 survey** | | |
| --- | --- | --- | --- |
|  | **No** | **Yes** | **Chi-squared p- value** |
| **Baseline Characteristics** | **N=855** | **N=1226** |  |
| **Sex** |  |  |  |
| Female | 451 (52.7) | 513 (41.8) | <0.001 |
| Male | 404 (47.3) | 713 (58.2) |  |
| **Age group** |  |  |  |
| 15-24 | 263 (30.8) | 219(17.9) | <0.001 |
| 25-34 | 297 (34.7) | 526 (42.9) |  |
| 35-44 | 207 (24.2) | 427 (34.8) |  |
| 45+ | 88 (10.3) | 54 (4.4) |  |
| **Marital status** |  |  |  |
| Married | 500 (58.5) | 769 (62.7) | 0.003 |
| Separated/Divorced | 218 (25.5) | 323 (26.3) |  |
| Never married | 137 (16) | 134 (10.9) |  |
| **Occupation** |  |  |  |
| Agriculture | 71 (8.3) | 90 (7.3) | <0.001 |
| Administrative | 26 (3) | 20 (1.6) |  |
| Trading | 185 (21.6) | 275 (22.4) |  |
| Fishing | 209 (24.4) | 421 (34.3) |  |
| Student | 26 (3) | 22 (1.8) |  |
| Unemployed | 118 (13.8) | 101 (8.2) |  |
| Others | 220 (25.7) | 297 (24.2) |  |
| **HIV risk group** |  |  |  |
| 0-50 | 30 (3.5) | 17 (1.4) | <0.001 |
| 51-100 | 176 (20.6) | 239 (19.5) |  |
| 101-150 | 343 (40.1) | 499 (40.7) |  |
| 151-200 | 225 (26.3) | 391 (31.9) |  |
| 201-250 | 65 (7.6) | 68 (5.5) |  |
| 251+ | 16 (1.9) | 12 (1) |  |
| **Number of sexual partners in past 12 months** |  |  |  |
| 0 to 1 partner | 537 (62.8) | 732 (59.7) | 0.154 |
| More than 1 partner | 318 (37.2) | 494 (40.3) |  |
| **Inconsistent condom use with non-marital partner** |  |  |  |
| No | 533 (62.3) | 716 (58.4) | 0.071 |
| Yes | 322 (37.7) | 510 (41.6) |  |
| **Transactional sex** |  |  |  |
| No | 548 (64.1) | 866 (70.6) | 0.002 |
| Yes | 307 (35.9) | 360 (29.4) |  |
| **HIV status** |  |  |  |
| Negative | 566 (66.2) | 774 (63.1) | 0.151 |
| Positive | 289 (33.8) | 452 (36.9) |  |
| **Household was in flooded area** |  |  |  |
| Yes | 353 (41.3) | 506 (41.3) | 0.995 |
| No | 502 (58.7) | 720 (58.7) |  |
