# Supplementary material for: Impact of natural disasters on HIV risk behaviors, seroprevalence, and virological supression in a hyperendemic fishing village in Uganda
Source: PLoS One. 2024 Oct 11;19(10):e0293711. doi: 10.1371/journal.pone.0293711 (PMC11469503; doi:10.1371/journal.pone.0293711)
Supplement: S4 Table — (DOCX) [file pone.0293711.s004.docx]

**S4 Table. Matched-pair conditional logistic regression analyses.**

|  | **Unadjusted** | | **Adjusted** | |
| --- | --- | --- | --- | --- |
| **Outcome** | **Odds Ratio (95% CI)** | **P value** | **Odds Ratio (95% CI)** | **P value** |
| **More than one sexual partner in the past 12 months** |  |  |  |  |
| Not flooded | 1 |  | 1 |  |
| Flooded | 1.05 (0.77-1.44) | 0.747 | 1.09 (0.77-1.54) | 0.625 |
| **Inconsistent condom use with non-marital partner** |  |  |  |  |
| Not flooded | 1 |  | 1 |  |
| Flooded | 0.74 (0.54-1.00) | 0.053 | 0.84 (0.57-1.23) | 0.37 |
| **Transactional sex** |  |  |  |  |
| Not flooded | 1 |  | 1 |  |
| Flooded | 0.62 (0.42-0.93) | 0.022 | 0.60 (0.38-0.95) | 0.03 |
