# Supplementary figures and images for: Impact of natural disasters on HIV risk behaviors, seroprevalence, and virological supression in a hyperendemic fishing village in Uganda
Source: PLoS One. 2024 Oct 11;19(10):e0293711. doi: 10.1371/journal.pone.0293711 (PMC11469503; doi:10.1371/journal.pone.0293711)

**S5 Figure.** **Spaghetti plot for HIV risk score, by gender, comparing flooding and COVID exposure.**


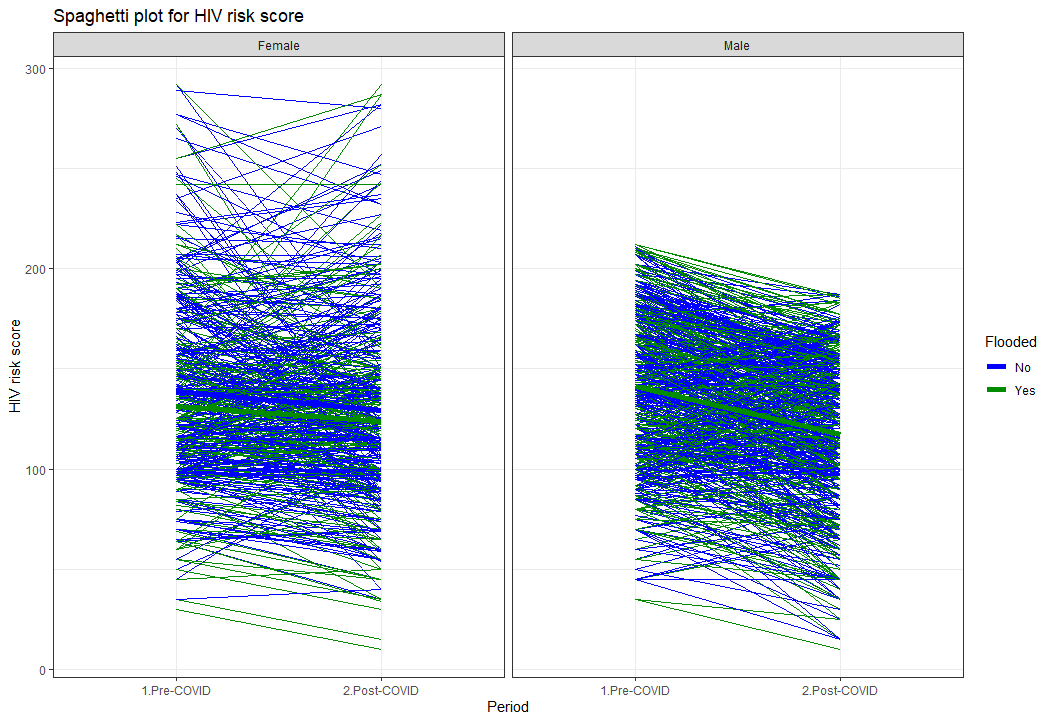

Supplement: S1 Fig — (DOCX) [file pone.0293711.s005.docx]
