# Supplementary material for: Impact of natural disasters on HIV risk behaviors, seroprevalence, and virological supression in a hyperendemic fishing village in Uganda
Source: PLoS One. 2024 Oct 11;19(10):e0293711. doi: 10.1371/journal.pone.0293711 (PMC11469503; doi:10.1371/journal.pone.0293711)
Supplement: Attachment — Submitted filename: COVID-Flooding_Responsetoreviewers_20240315.docx [file pone.0293711.s006.docx]

May 24, 2024

To Xiaoyu Song, Academic Editor,

Thank you for the positive review of our manuscript, *“Impact of natural disasters on HIV risk behaviors, seroprevalence, and virological supression in a hyperendemic fishing village in Uganda”* [PONE-D-23-33428] for consideration by *PLOS ONE.*

Below, we have included the comments from the reviewer and the academic editor and how we have responded to each in blue. We appreciate the comments from both reviewers and believe they have strengthened the manuscript. We hope that these revisions address the points raised during the review process and that you will accept this manuscript for publication.

Sincerely,

Hadijja Nakawooya

Statistician

Rakai Health Sciences Program

Kalisizo, Uganda

**Reviewer 1**

The manuscript used the data from an open longitudinal population-based HIV surveillance cohort in Lake Victoria fishing communities, which were affected by severe flooding and COVID-19 at the same time, to assess the changes in HIV-associated behaviors and treatment outcomes before and after COVID-19 among flooded and non-flooded households. To address the research questions, a difference-in-differences analysis was employed using a modified Poisson regression model. While the manuscript generally succeeds in describing the research questions, conducting statistical analyses, and presenting results adequately, there are still a lot of improvements needed for publication purposes. Specifically, there is a need for a more detailed elaboration on the background and purpose of the research study. Consideration could be given to employing a better model which accounts for the within-subject correlation. Additionally, several suggestions for correcting errors in the results section have been provided in the comments below.

Thank you for this generally positive review.

Major comments:

1. The author should provide further justifications for the research objective and the reason of choosing population in Lake Victoria fishing communities as study population. While it is intuitive to recognize the importance of studying the impacts of floods or COVID lockdown on HIV separately, readers may not immediately grasp why understanding the integrated effects of flooding and COVID-19 on HIV is essential.

We appreciate the reviewer's valuable input. As per their suggestion, we have made additions and modifications to the sentences in the first paragraph of the introduction, elucidating the rationale behind selecting the population from Lake Victoria fishing communities as our study sample: “Lake Victoria fishing communities in eastern Africa have one of the highest HIV burdens globally, with adult HIV seroprevalence estimates ranging from 20 to 40% and HIV transmission remains high with incidence of 1.59/100 per person years [10, 11]. Many people in this fishing community engage in high-risk sexual behaviors and drug and alcohol use. Women frequently report risky sex behaviors which is often related to sex and bar work. The situation is further complicated with increased vulnerability to HIV, including increased peer pressure especially among adolescents and poor housing comprised of make-shift shelters made of timber and occupied by two or more families of different backgrounds and their children.”

We have also made additions and modifications to the sentences in the second paragraph of the introduction, elucidating the rationale of studying the impacts of floods or COVID lockdown on HIV separately: “Floods disrupt lives and health practices, raising HIV transmission risks due to behavior changes and limited prevention resources[9, 17]. Reduced support for individuals with HIV, coupled with limited information on HIV and the disruption of counseling and testing services by floods, contributes to undiagnosed cases and unsafe sex practices[18]. Malnutrition and disease weaken immune systems, increasing virus transmission likelihood[7, 19]”..”

2. While the modified Poisson regression model is suitable for addressing the research questions, the author should offer justifications for choosing this model over other commonly used models, such as the log-binomial model. Furthermore, it is essential for the author to cite the method paper of the modified Poisson regression to provide proper reference in the statistical analysis section.

We thank the reviewer for this important comment. Two citations have been added to support the use of the modified Poisson regression in the statistical analysis section:

Chen, W., et al., *Comparison of robustness to outliers between robust poisson models and log-binomial models when estimating relative risks for common binary outcomes: a simulation study.* BMC Medical Research Methodology, 2014. **14**(1): p. 82.

Gallis, J.A. and E.L. Turner, *Relative Measures of Association for Binary Outcomes: Challenges and Recommendations for the Global Health Researcher.* Ann Glob Health, 2019. **85**(1): p. 137

3. Since the samples can be perfectly matched in the pre- and post-COVID-19 surveys, the author can consider using DID model with random effects or generalized estimating equation (GEE) to account for the within-subject correlation.

Thank for this excellent suggestion. We have now used a DID model with random effects applied to participant id to account for the within-subject correlation and updated our methods section (“The assessment of difference-in-differences (DID) was conducted by examining the coefficient of the interaction term between these variables, with random effects analysis applied to participant ID to account for the within-subject correlation.”) and results accordingly.

4. The manuscript needs editing for language and writing quality. The language is sometimes difficult to follow. Additionally, there are several typos in the manuscripts. One of the major ones is “difference in difference analysis” should be “difference-in-differences analysis”. The author should correct the typos and revise the language to improve readability, especially for the introduction section.

Thank you for catching this. We have adjusted our language to say “difference-in-differences analysis” and have gone through the entire manuscript again for readability.

Minor comments:

1. The author should employ clearer language when describing the primary outcomes in the analysis, ensuring that readers can easily distinguish the variable type, such as binary or count data. For instance, in the first sentence of the primary outcomes section, changing "inconsistent condom use" to "the frequency of inconsistent condom use" or "whether inconsistent condom use occurs" would clarify that it is a binary or count data, aiding readers in better understanding.

Thank you for catching this. We have edited our phrasing of the condom use outcome to say "whether inconsistent condom use occurs", so that it is clear to readers that this is a binary variable.

2. Since supplementary table 4 was mentioned many times in the results section, the author should consider moving the table to the main text instead of placing it in the supplementary information.

Thank you for this feedback. Supplementary Table 4 has been relocated to the main text and renamed as Table 2, and we have renumbered the subsequent tables.

3. In figure 3, the error bars are not fully represented in sub-figures C and F. For figure 3 and 4, there seems to be no clear rationale for stratifying by gender, especially considering that gender is not a variable of interest, and there is no significant difference in gender distribution as indicated in Table 1a.

Thank you for noticing this – we have edited the figure to ensure that the error bars are fully presented in each of the six plots. We chose to stratify by gender because men and women in this community have historically demonstrated different risk behaviors and reported different HIV outcomes.

4. In the results section, it was said “we observed significant declines in inconsistent condom use with non-marital partners and self-reported transactional sex, irrespective of household flooding status (Table 1b, Figure 3)”. Table 1b and figure 3 are not related to the conclusion.

Thank you for the catch; we had switched the table and figure – we have adjusted this to cite figure 3 and (what is now) table 3.

5. In table 2 and supplementary table 5, “1” should be changed to “Reference”.

We have made this change.

6. In the last paragraph of results section, the interpretations of DID model results are not accurate. “No significant differences” should be changed to “no significant changes in differences”.

We have made this change.

7. The author is aware that there are significant differences between participants included in the final analysis and participants lost to follow-up as indicated in supplementary table 3 but did not mention that the selection method utilized in the study could cause selection bias in the limitation of study.

Thank you for this comment. We have added this to the study limitations “Second, there was selection bias among respondents, as the study focused on individuals who stayed in the community both before and after flooding occurred; these participants differed from those who migrated out of the community, potentially due to being more affected by the flooding, thus leading to their migration.”

8. The author should elaborate more on how the results of the study will help inform public health policies and improve population health in the future in the discussion section.

We have added elaboration on the public health implications of this study to the discussion section: “In conclusion, despite a high background burden of HIV, the COVID-19 pandemic, and serious flooding, we observed no adverse impact on HIV risk behaviors, burden, or virological outcomes in this HIV hyperendemic fishing community. These findings highlights the importance of integrating HIV programming into disaster response efforts, which can inform customized interventions to lessen the impact of natural disasters on HIV. The resilience observed in HIV outcomes during crises underscores the significance of adaptable health systems, providing valuable insights to reinforce resilience in resource-limited settings. Innovations such as telemedicine and community-led ART delivery are pivotal in sustaining HIV care during emergencies. By incorporating these strategies into regular programming, access to services can be improved, especially for hard-to-reach populations. Understanding exactly what HIV programs and personal/community-level strategies worked to maintain good public health outcomes despite extreme environmental and pandemic conditions may help improve population health.”

**Editor**

The revised version should explain the rational or hypothesis for studying flooding and COVID-19 for HIV in the introduction and elucidate the public health implications of this study. Additionally, the revision ought to address all the statistical issues raised by the reviewer, ensure coherence between tables/figures and the narrative, and enhance the overall readability of the manuscript.

Thank you for these overarching comments. We have added the rationale for examining the impact of COVID and flooding on HIV to the introduction, and have elucidated the public health implications of this study in the discussion section. We have also addressed the statistical issues raised by the reviewer (see responses above), ensured coherence between the tables/figures and the narrative, and doubled checked the overall readability of the manuscript.

Journal Requirements:

When submitting your revision, we need you to address these additional requirements.

1. Please ensure that your manuscript meets PLOS ONE's style requirements, including those for file naming. The PLOS ONE style templates can be found at 
[https://journals.plos.org/plosone/s/file?id=wjVg/PLOSOne_formatting_sample_main_body.pdf](https://nam02.safelinks.protection.outlook.com/?url=https%3A%2F%2Fjournals.plos.org%2Fplosone%2Fs%2Ffile%3Fid%3DwjVg%2FPLOSOne_formatting_sample_main_body.pdf&data=05%7C02%7Cteresa.yeh%40jhu.edu%7Cb3718442a7d042a5248e08dc4363fee0%7C9fa4f438b1e6473b803f86f8aedf0dec%7C0%7C0%7C638459347141962589%7CUnknown%7CTWFpbGZsb3d8eyJWIjoiMC4wLjAwMDAiLCJQIjoiV2luMzIiLCJBTiI6Ik1haWwiLCJXVCI6Mn0%3D%7C0%7C%7C%7C&sdata=GKfKbZzVx%2FDFViLeQMRkClip8gwnn7CWcaQKcYimU08%3D&reserved=0) and 
[https://journals.plos.org/plosone/s/file?id=ba62/PLOSOne_formatting_sample_title_authors_affiliations.pdf](https://nam02.safelinks.protection.outlook.com/?url=https%3A%2F%2Fjournals.plos.org%2Fplosone%2Fs%2Ffile%3Fid%3Dba62%2FPLOSOne_formatting_sample_title_authors_affiliations.pdf&data=05%7C02%7Cteresa.yeh%40jhu.edu%7Cb3718442a7d042a5248e08dc4363fee0%7C9fa4f438b1e6473b803f86f8aedf0dec%7C0%7C0%7C638459347141969392%7CUnknown%7CTWFpbGZsb3d8eyJWIjoiMC4wLjAwMDAiLCJQIjoiV2luMzIiLCJBTiI6Ik1haWwiLCJXVCI6Mn0%3D%7C0%7C%7C%7C&sdata=t4XurCvDsSmy0Lb5UAtJksSXFT6KvpIfrDeK2zhxzVw%3D&reserved=0)

We have doubled-checked style requirements per the style templates.

2. Please include a complete copy of PLOS’ questionnaire on inclusivity in global research in your revised manuscript. Our policy for research in this area aims to improve transparency in the reporting of research performed outside of researchers’ own country or community. The policy applies to researchers who have travelled to a different country to conduct research, research with Indigenous populations or their lands, and research on cultural artefacts. The questionnaire can also be requested at the journal’s discretion for any other submissions, even if these conditions are not met.  Please find more information on the policy and a link to download a blank copy of the questionnaire here: [https://journals.plos.org/plosone/s/best-practices-in-research-reporting](https://nam02.safelinks.protection.outlook.com/?url=https%3A%2F%2Fjournals.plos.org%2Fplosone%2Fs%2Fbest-practices-in-research-reporting&data=05%7C02%7Cteresa.yeh%40jhu.edu%7Cb3718442a7d042a5248e08dc4363fee0%7C9fa4f438b1e6473b803f86f8aedf0dec%7C0%7C0%7C638459347141974859%7CUnknown%7CTWFpbGZsb3d8eyJWIjoiMC4wLjAwMDAiLCJQIjoiV2luMzIiLCJBTiI6Ik1haWwiLCJXVCI6Mn0%3D%7C0%7C%7C%7C&sdata=4yBUs4oN87qh%2BWHb0R7wwaCwloBImzm5yrAmLY86KUA%3D&reserved=0). Please upload a completed version of your questionnaire as Supporting Information when you resubmit your manuscript.

We have completed this questionnaire and uploaded it as supporting information.

3. Please note that funding information should not appear in any section or other areas of your manuscript. We will only publish funding information present in the Funding Statement section of the online submission form. Please remove any funding-related text from the manuscript.

We have removed all funding-related text in the manuscript.

4. In this instance it seems there may be acceptable restrictions in place that prevent the public sharing of your minimal data. However, in line with our goal of ensuring long-term data availability to all interested researchers, PLOS’ Data Policy states that authors cannot be the sole named individuals responsible for ensuring data access ([http://journals.plos.org/plosone/s/data-availability#loc-acceptable-data-sharing-methods](https://nam02.safelinks.protection.outlook.com/?url=http%3A%2F%2Fjournals.plos.org%2Fplosone%2Fs%2Fdata-availability%23loc-acceptable-data-sharing-methods&data=05%7C02%7Cteresa.yeh%40jhu.edu%7Cb3718442a7d042a5248e08dc4363fee0%7C9fa4f438b1e6473b803f86f8aedf0dec%7C0%7C0%7C638459347141980640%7CUnknown%7CTWFpbGZsb3d8eyJWIjoiMC4wLjAwMDAiLCJQIjoiV2luMzIiLCJBTiI6Ik1haWwiLCJXVCI6Mn0%3D%7C0%7C%7C%7C&sdata=7xTdBqY%2BwjiCAOVNQLWhd703IiSPPz%2Fo80dm8lXatlA%3D&reserved=0)).

Data requests to a non-author institutional point of contact, such as a data access or ethics committee, helps guarantee long term stability and availability of data. Providing interested researchers with a durable point of contact ensures data will be accessible even if an author changes email addresses, institutions, or becomes unavailable to answer requests.

Before we proceed with your manuscript, please also provide non-author contact information (phone/email/hyperlink) for a data access committee, ethics committee, or other institutional body to which data requests may be sent. If no institutional body is available to respond to requests for your minimal data, please consider if there any institutional representatives who did not collaborate in the study, and are not listed as authors on the manuscript, who would be able to hold the data and respond to external requests for data access? If so, please provide their contact information (i.e., email address). Please also provide details on how you will ensure persistent or long-term data storage and availability.

We appreciate the effort that the PLOS journal family takes to make data available (and to guarantee long-term stability and availability of data despite potential changes to the institutional affiliations etc of individual authors). Data requests may be sent to the Rakai Health Sciences Program data management office (datarequests@rhsp.org), where data are archived across all the various projects run by the RHSP (original paper forms from the RCCS surveys, as well as the electronic datasets for each survey round).

5. PLOS requires an ORCID iD for the corresponding author in Editorial Manager on papers submitted after December 6th, 2016. Please ensure that you have an ORCID iD and that it is validated in Editorial Manager. To do this, go to ‘Update my Information’ (in the upper left-hand corner of the main menu), and click on the Fetch/Validate link next to the ORCID field. This will take you to the ORCID site and allow you to create a new iD or authenticate a pre-existing iD in Editorial Manager. Please see the following video for instructions on linking an ORCID iD to your Editorial Manager account: [https://www.youtube.com/watch?v=_xcclfuvtxQ](https://nam02.safelinks.protection.outlook.com/?url=https%3A%2F%2Fwww.youtube.com%2Fwatch%3Fv%3D_xcclfuvtxQ&data=05%7C02%7Cteresa.yeh%40jhu.edu%7Cb3718442a7d042a5248e08dc4363fee0%7C9fa4f438b1e6473b803f86f8aedf0dec%7C0%7C0%7C638459347141986250%7CUnknown%7CTWFpbGZsb3d8eyJWIjoiMC4wLjAwMDAiLCJQIjoiV2luMzIiLCJBTiI6Ik1haWwiLCJXVCI6Mn0%3D%7C0%7C%7C%7C&sdata=GaLBg7NUSTnW07yU11lQUKRx4l96THYOk8BES9YnnGo%3D&reserved=0)

We have inserted the ORCID iD for the corresponding author: 0009-0006-5430-3009

6. We note that Figure 2 in your submission contain map/satellite images which may be copyrighted. All PLOS content is published under the Creative Commons Attribution License (CC BY 4.0), which means that the manuscript, images, and Supporting Information files will be freely available online, and any third party is permitted to access, download, copy, distribute, and use these materials in any way, even commercially, with proper attribution. For these reasons, we cannot publish previously copyrighted maps or satellite images created using proprietary data, such as Google software (Google Maps, Street View, and Earth). For more information, see our copyright guidelines: [http://journals.plos.org/plosone/s/licenses-and-copyright](https://nam02.safelinks.protection.outlook.com/?url=http%3A%2F%2Fjournals.plos.org%2Fplosone%2Fs%2Flicenses-and-copyright&data=05%7C02%7Cteresa.yeh%40jhu.edu%7Cb3718442a7d042a5248e08dc4363fee0%7C9fa4f438b1e6473b803f86f8aedf0dec%7C0%7C0%7C638459347141991449%7CUnknown%7CTWFpbGZsb3d8eyJWIjoiMC4wLjAwMDAiLCJQIjoiV2luMzIiLCJBTiI6Ik1haWwiLCJXVCI6Mn0%3D%7C0%7C%7C%7C&sdata=1K3CV%2Bw914SFYW40lg3wPEEHXW2hd%2FrwWkDoDzGTBXs%3D&reserved=0).
 
We require you to either (a) present written permission from the copyright holder to publish these figures specifically under the CC BY 4.0 license, or (b) remove the figures from your submission:
 
a. You may seek permission from the original copyright holder of Figure 2 to publish the content specifically under the CC BY 4.0 license.  
 
We recommend that you contact the original copyright holder with the Content Permission Form ([http://journals.plos.org/plosone/s/file?id=7c09/content-permission-form.pdf](https://nam02.safelinks.protection.outlook.com/?url=http%3A%2F%2Fjournals.plos.org%2Fplosone%2Fs%2Ffile%3Fid%3D7c09%2Fcontent-permission-form.pdf&data=05%7C02%7Cteresa.yeh%40jhu.edu%7Cb3718442a7d042a5248e08dc4363fee0%7C9fa4f438b1e6473b803f86f8aedf0dec%7C0%7C0%7C638459347141996846%7CUnknown%7CTWFpbGZsb3d8eyJWIjoiMC4wLjAwMDAiLCJQIjoiV2luMzIiLCJBTiI6Ik1haWwiLCJXVCI6Mn0%3D%7C0%7C%7C%7C&sdata=j7VPHFLvumlU5GLt04lki2ZP2O3u3jpVfKKOWJ82djc%3D&reserved=0)) and the following text:
“I request permission for the open-access journal PLOS ONE to publish XXX under the Creative Commons Attribution License (CCAL) CC BY 4.0 ([http://creativecommons.org/licenses/by/4.0/](https://nam02.safelinks.protection.outlook.com/?url=http%3A%2F%2Fcreativecommons.org%2Flicenses%2Fby%2F4.0%2F&data=05%7C02%7Cteresa.yeh%40jhu.edu%7Cb3718442a7d042a5248e08dc4363fee0%7C9fa4f438b1e6473b803f86f8aedf0dec%7C0%7C0%7C638459347142002200%7CUnknown%7CTWFpbGZsb3d8eyJWIjoiMC4wLjAwMDAiLCJQIjoiV2luMzIiLCJBTiI6Ik1haWwiLCJXVCI6Mn0%3D%7C0%7C%7C%7C&sdata=5zukuTyF1oplNYAbhdU0RJ0mmOEDpRZ5nPliA60d6ys%3D&reserved=0)). Please be aware that this license allows unrestricted use and distribution, even commercially, by third parties. Please reply and provide explicit written permission to publish XXX under a CC BY license and complete the attached form.”
 
Please upload the completed Content Permission Form or other proof of granted permissions as an "Other" file with your submission.
 
In the figure caption of the copyrighted figure, please include the following text: “Reprinted from [ref] under a CC BY license, with permission from [name of publisher], original copyright [original copyright year].”
 
b. If you are unable to obtain permission from the original copyright holder to publish these figures under the CC BY 4.0 license or if the copyright holder’s requirements are incompatible with the CC BY 4.0 license, please either i) remove the figure or ii) supply a replacement figure that complies with the CC BY 4.0 license. Please check copyright information on all replacement figures and update the figure caption with source information. If applicable, please specify in the figure caption text when a figure is similar but not identical to the original image and is therefore for illustrative purposes only.
The following resources for replacing copyrighted map figures may be helpful:

USGS National Map Viewer (public domain): [http://viewer.nationalmap.gov/viewer/](https://nam02.safelinks.protection.outlook.com/?url=http%3A%2F%2Fviewer.nationalmap.gov%2Fviewer%2F&data=05%7C02%7Cteresa.yeh%40jhu.edu%7Cb3718442a7d042a5248e08dc4363fee0%7C9fa4f438b1e6473b803f86f8aedf0dec%7C0%7C0%7C638459347142007566%7CUnknown%7CTWFpbGZsb3d8eyJWIjoiMC4wLjAwMDAiLCJQIjoiV2luMzIiLCJBTiI6Ik1haWwiLCJXVCI6Mn0%3D%7C0%7C%7C%7C&sdata=%2Bezxb0RHEckdCoHJ2V9Nu012UJMuGbD8MZ88L2ojXG8%3D&reserved=0)
The Gateway to Astronaut Photography of Earth (public domain): [http://eol.jsc.nasa.gov/sseop/clickmap/](https://nam02.safelinks.protection.outlook.com/?url=http%3A%2F%2Feol.jsc.nasa.gov%2Fsseop%2Fclickmap%2F&data=05%7C02%7Cteresa.yeh%40jhu.edu%7Cb3718442a7d042a5248e08dc4363fee0%7C9fa4f438b1e6473b803f86f8aedf0dec%7C0%7C0%7C638459347142012787%7CUnknown%7CTWFpbGZsb3d8eyJWIjoiMC4wLjAwMDAiLCJQIjoiV2luMzIiLCJBTiI6Ik1haWwiLCJXVCI6Mn0%3D%7C0%7C%7C%7C&sdata=j3IBn4QtUdc5USwyaxt8NxG90ThrexBUl0H3moNsYi8%3D&reserved=0)
Maps at the CIA (public domain): [https://www.cia.gov/library/publications/the-world-factbook/index.html](https://nam02.safelinks.protection.outlook.com/?url=https%3A%2F%2Fwww.cia.gov%2Flibrary%2Fpublications%2Fthe-world-factbook%2Findex.html&data=05%7C02%7Cteresa.yeh%40jhu.edu%7Cb3718442a7d042a5248e08dc4363fee0%7C9fa4f438b1e6473b803f86f8aedf0dec%7C0%7C0%7C638459347142017853%7CUnknown%7CTWFpbGZsb3d8eyJWIjoiMC4wLjAwMDAiLCJQIjoiV2luMzIiLCJBTiI6Ik1haWwiLCJXVCI6Mn0%3D%7C0%7C%7C%7C&sdata=Nx5p09PRXWwOeO6gnyOe1D8l8I97zl2GE1x4rNr5DFA%3D&reserved=0) and [https://www.cia.gov/library/publications/cia-maps-publications/index.html](https://nam02.safelinks.protection.outlook.com/?url=https%3A%2F%2Fwww.cia.gov%2Flibrary%2Fpublications%2Fcia-maps-publications%2Findex.html&data=05%7C02%7Cteresa.yeh%40jhu.edu%7Cb3718442a7d042a5248e08dc4363fee0%7C9fa4f438b1e6473b803f86f8aedf0dec%7C0%7C0%7C638459347142023107%7CUnknown%7CTWFpbGZsb3d8eyJWIjoiMC4wLjAwMDAiLCJQIjoiV2luMzIiLCJBTiI6Ik1haWwiLCJXVCI6Mn0%3D%7C0%7C%7C%7C&sdata=x9hHG5y%2Fydwt90aututHSdYoGjvkD9POTYtzTzSPh6I%3D&reserved=0)
NASA Earth Observatory (public domain): [http://earthobservatory.nasa.gov/](https://nam02.safelinks.protection.outlook.com/?url=http%3A%2F%2Fearthobservatory.nasa.gov%2F&data=05%7C02%7Cteresa.yeh%40jhu.edu%7Cb3718442a7d042a5248e08dc4363fee0%7C9fa4f438b1e6473b803f86f8aedf0dec%7C0%7C0%7C638459347142028267%7CUnknown%7CTWFpbGZsb3d8eyJWIjoiMC4wLjAwMDAiLCJQIjoiV2luMzIiLCJBTiI6Ik1haWwiLCJXVCI6Mn0%3D%7C0%7C%7C%7C&sdata=veLGDaWBQE9h99Eci8YQRtBMdGEiFsLVD0AnjhqcB4U%3D&reserved=0)
Landsat: [http://landsat.visibleearth.nasa.gov/](https://nam02.safelinks.protection.outlook.com/?url=http%3A%2F%2Flandsat.visibleearth.nasa.gov%2F&data=05%7C02%7Cteresa.yeh%40jhu.edu%7Cb3718442a7d042a5248e08dc4363fee0%7C9fa4f438b1e6473b803f86f8aedf0dec%7C0%7C0%7C638459347142033235%7CUnknown%7CTWFpbGZsb3d8eyJWIjoiMC4wLjAwMDAiLCJQIjoiV2luMzIiLCJBTiI6Ik1haWwiLCJXVCI6Mn0%3D%7C0%7C%7C%7C&sdata=tf8sr04XB4GM2hHPagah9XDZdY491Iyi3i90rJ%2FNGOs%3D&reserved=0)
USGS EROS (Earth Resources Observatory and Science (EROS) Center) (public domain): [http://eros.usgs.gov/#](https://nam02.safelinks.protection.outlook.com/?url=http%3A%2F%2Feros.usgs.gov%2F&data=05%7C02%7Cteresa.yeh%40jhu.edu%7Cb3718442a7d042a5248e08dc4363fee0%7C9fa4f438b1e6473b803f86f8aedf0dec%7C0%7C0%7C638459347142038197%7CUnknown%7CTWFpbGZsb3d8eyJWIjoiMC4wLjAwMDAiLCJQIjoiV2luMzIiLCJBTiI6Ik1haWwiLCJXVCI6Mn0%3D%7C0%7C%7C%7C&sdata=tWMBjc6rG7ltzaJqjMLyEO3UXEYuun2aXd1uiuI6CiE%3D&reserved=0)
Natural Earth (public domain): [http://www.naturalearthdata.com/](https://nam02.safelinks.protection.outlook.com/?url=http%3A%2F%2Fwww.naturalearthdata.com%2F&data=05%7C02%7Cteresa.yeh%40jhu.edu%7Cb3718442a7d042a5248e08dc4363fee0%7C9fa4f438b1e6473b803f86f8aedf0dec%7C0%7C0%7C638459347142043208%7CUnknown%7CTWFpbGZsb3d8eyJWIjoiMC4wLjAwMDAiLCJQIjoiV2luMzIiLCJBTiI6Ik1haWwiLCJXVCI6Mn0%3D%7C0%7C%7C%7C&sdata=BrRldJm35JDSkOLbpS2mjUBgFUeOJ9MteEzUEoO5srM%3D&reserved=0)

For Figure 2, we have now noted in the figure caption that this was adapted from open source shapefiles and cited the original sources:

Uganda Bureau of Statistics. (2012). Lakes and Rivers: Uganda, 2005-2006. [Shapefile]. Uganda Bureau of Statistics. Retrieved from <https://earthworks.stanford.edu/catalog/stanford-fh022bz4757>

Uganda Bureau of Statistics (2012) Sub-County Boundaries, Uganda, 2010. Uganda Bureau of Statistics Available at: <http://purl.stanford.edu/yg674hb9000>

7. Please include captions for your Supporting Information files at the end of your manuscript, and update any in-text citations to match accordingly. Please see our Supporting Information guidelines for more information: [http://journals.plos.org/plosone/s/supporting-information](https://nam02.safelinks.protection.outlook.com/?url=http%3A%2F%2Fjournals.plos.org%2Fplosone%2Fs%2Fsupporting-information&data=05%7C02%7Cteresa.yeh%40jhu.edu%7Cb3718442a7d042a5248e08dc4363fee0%7C9fa4f438b1e6473b803f86f8aedf0dec%7C0%7C0%7C638459347142048161%7CUnknown%7CTWFpbGZsb3d8eyJWIjoiMC4wLjAwMDAiLCJQIjoiV2luMzIiLCJBTiI6Ik1haWwiLCJXVCI6Mn0%3D%7C0%7C%7C%7C&sdata=IqecyaxFf8R9A7Tvuqy5y6qyTUwupeZjf5PNpvgb4iM%3D&reserved=0).

We have added captions to the supporting information files and updated in-text citations accordingly.
