# Supplementary material for: Impact of natural disasters on HIV risk behaviors, seroprevalence, and virological supression in a hyperendemic fishing village in Uganda
Source: PLoS One. 2024 Oct 11;19(10):e0293711. doi: 10.1371/journal.pone.0293711 (PMC11469503; doi:10.1371/journal.pone.0293711)
Supplement: Attachment — Submitted filename: Reviewers.docx [file pone.0293711.s007.docx]

Reviewers' comments:

Reviewer's Responses to Questions

**Comments to the Author**

1. If the authors have adequately addressed your comments raised in a previous round of review and you feel that this manuscript is now acceptable for publication, you may indicate that here to bypass the “Comments to the Author” section, enter your conflict of interest statement in the “Confidential to Editor” section, and submit your "Accept" recommendation.

Reviewer #1: (No Response)

2. Is the manuscript technically sound, and do the data support the conclusions?

The manuscript must describe a technically sound piece of scientific research with data that supports the conclusions. Experiments must have been conducted rigorously, with appropriate controls, replication, and sample sizes. The conclusions must be drawn appropriately based on the data presented.

Reviewer #1: Yes

3. Has the statistical analysis been performed appropriately and rigorously?

Reviewer #1: Yes

4. Have the authors made all data underlying the findings in their manuscript fully available?

The [PLOS Data policy](http://www.plosone.org/static/policies.action#sharing) requires authors to make all data underlying the findings described in their manuscript fully available without restriction, with rare exception (please refer to the Data Availability Statement in the manuscript PDF file). The data should be provided as part of the manuscript or its supporting information, or deposited to a public repository. For example, in addition to summary statistics, the data points behind means, medians and variance measures should be available. If there are restrictions on publicly sharing data—e.g. participant privacy or use of data from a third party—those must be specified.

Reviewer #1: (No Response)

Yes the data is available after completing a data request form.

5. Is the manuscript presented in an intelligible fashion and written in standard English?

PLOS ONE does not copyedit accepted manuscripts, so the language in submitted articles must be clear, correct, and unambiguous. Any typographical or grammatical errors should be corrected at revision, so please note any specific errors here.

Reviewer #1: (No Response)

All the typographical or grammatical errors have been corrected.

6. Review Comments to the Author

Please use the space provided to explain your answers to the questions above. You may also include additional comments for the author, including concerns about dual publication, research ethics, or publication ethics. (Please upload your review as an attachment if it exceeds 20,000 characters)

Reviewer #1: The manuscript utilized data from Lake Victoria fishing communities, which were simultaneously affected by severe flooding and COVID-19, to assess changes in HIV-associated behaviors and treatment outcomes before and after COVID-19 among flooded and non-flooded households. In the initial submission, requests were made for a more detailed elaboration on the rationale and purpose of the study, as well as corrections to the results section.

Major revisions:

1. In the introduction section of the manuscript, the author elaborated more on the rationale for using data from fishing communities and studying the impacts of floods on HIV. This addition addressed the reviewers' and editors' requests for a more detailed explanation of the rationale or hypothesis behind investigating the relationship between flooding and HIV.
2. For statistical modeling, the DID model now included random effects to account for the within-subject correlation. Additionally, the author included two references for the statistical model used and revised the wording of the variables in the analysis to clarify their types.
3. In the discussion section, potential selection bias was addressed as one of the limitations of the study. Additionally, further elaborations on the public health implications of the study were included.


Minor revisions:
1. In the results section, the author addressed the issues pointed out by the reviewer and made changes/corrections accordingly, such as changing the phrasing of variables, editing the figures and moving table S4 to main text.

In general, the re-submitted manuscript addressed the issues pointed out by the reviewer and editor properly. However, the readability can still be improved by refining the wording, improving the logical flow, and considering restructuring parts of the manuscript. Additionally, there are still typos in the re-submitted manuscript, for example, the first sentence of the fourth paragraph in the introduction section: “we conducted and pre and post study to evaluate the impact of COVID-19 and flooding on …”.
Addressing these issues will help ensure that your valuable research is communicated as effectively as possible to readers and meet the journal's standards.

7. PLOS authors have the option to publish the peer review history of their article ([what does this mean?](https://journals.plos.org/plosone/s/editorial-and-peer-review-process#loc-peer-review-history)). If published, this will include your full peer review and any attached files.


If you choose “no”, your identity will remain anonymous but your review may still be made public.


**Do you want your identity to be public for this peer review?** For information about this choice, including consent withdrawal, please see our [Privacy Policy](https://www.plos.org/privacy-policy).

Reviewer #1: **Yes:** Weijia Fu

[NOTE: If reviewer comments were submitted as an attachment file, they will be attached to this email and accessible via the submission site. Please log into your account, locate the manuscript record, and check for the action link "View Attachments". If this link does not appear, there are no attachment files.]

While revising your submission, please upload your figure files to the Preflight Analysis and Conversion Engine (PACE) digital diagnostic tool, <https://pacev2.apexcovantage.com/>. PACE helps ensure that figures meet PLOS requirements. To use PACE, you must first register as a user. Registration is free. Then, login and navigate to the UPLOAD tab, where you will find detailed instructions on how to use the tool. If you encounter any issues or have any questions when using PACE, please email PLOS at [figures@plos.org](mailto:figures@plos.org). Please note that Supporting Information files do not need this step.

*In compliance with data protection regulations, you may request that we remove your personal registration details at any time.* [*(Remove my information/details)*](https://www.editorialmanager.com/pone/login.asp?a=r)*. Please contact the publication office if you have any questions.*
